# The Preparation of a GO/ZnO/nHAp Composite Coating and the Study of Its Performance Optimization for Pure Titanium Implants

**DOI:** 10.3390/mi16060637

**Published:** 2025-05-28

**Authors:** Jiang Wu, Yu Zuo, Zhaoxi Xu, Lang Wang, Jiaju Zou, Zijian Jia, Chunmei Wang, Guoliang Zhang

**Affiliations:** School of Stomatology, Jiamusi University, Jiamusi 154007, China; wujiangwj0126@163.com (J.W.); zhangsszss2@163.com (Y.Z.); 13603693279@163.com (Z.X.); 13359555272@163.com (L.W.); 17752712734@163.com (J.Z.); aaa643807758@163.com (Z.J.); wcm13603693279@163.com (C.W.)

**Keywords:** corrosion resistance, bone guidance, hydrothermal technology, GO/ZnO/nHAp composite coating, microarc oxidation

## Abstract

In this study, a graphene oxide (GO)/zinc oxide (ZnO)/hydroxyapatite (nHAp) composite coating was constructed on a pure titanium surface by microarc oxidation (MAO) pretreatment combined with hydrothermal technology (HT), thereby making it possible to explore the performance optimization of this coating for Ti-based implants. Scanning electron microscopy (SEM), an energy dispersion spectrometer (EDS), Fourier transform infrared spectroscopy (FTIR), Ramam spectroscopy (Ramam), etc., confirmed that the GO/ZnO/nHAp composites were successfully loaded onto the pure Ti surfaces. Through nanoindentation, differential thermal analysis (DiamondTG/DTA), and dynamic polarization potential detection, the GO/ZnO/nHAp composite coating imparts excellent nanohardness (2.7 + 1.0 GPa), elastic modulus (53.5 + 1.0 GPa), thermal stability, and corrosion resistance to pure Ti implants; hemolysis rate analysis, CCK-8, alkaline phosphatase (ALP) detection, alizarin red staining, and other experiments further show that the coating improves the hemocompatibility, biocompatibility, and bone guidance of the Ti implant surface. Studies have shown that GO/ZnO/nHAp composite coatings can effectively optimize the mechanical properties, corrosion resistance, biocompatibility, and bone guidance of pure Ti implants, so that they can obtain an elastic modulus that matches human bone.

## 1. Introduction

At present, titanium and its alloys are widely used in bone tissue engineering as metal materials in the field of the biomedical sciences because of their high specific strength and low cytotoxicity [1,2,3]. However, since the elastic modulus of titanium is higher than that of cortical bone (7–30 GPa) and cancellous bone (0.1–0.5 GPa) [4], when using it as a base implant, stress shielding problems will be caused by the mismatch of elastic moduli [5]. In addition, studies have found that metal elements in implant materials can lead to corrosion-related biological problems if discharged into tissues around the surgical area [6]. Therefore, this study intends to modify the titanium surface, aiming to obtain an elastic modulus that matches human bone, and enhance the mechanical properties such as the corrosion resistance and biological activity of the implant using a coating, thereby improving the success rate of the implant.

Hydroxyapatite (Ca_10_ (PO_4_)_6_(OH)_2_, HAp) is often used in orthopedics, dentistry, and other medical applications. Since hydroxyapatite is the main inorganic component of human bone tissue [7], it has the advantages of low cost, simple preparation, and good compatibility with the human body’s tissue engineering scaffolds [8], drug carriers [9], hydrogels [10], and other aspects. However, HAp has poor biomechanical properties, such as high brittleness and low fracture toughness. Therefore, the mechanical properties of HAp are improved by combining it with other materials [11,12].

Graphene oxide (GO) sheets are becoming one of the most important graphene derivatives for biomedical applications [13], and are used in cardiac tissue engineering [14], biosensors [15], bioimaging [16], auxiliary diagnosis [17], and other aspects. Graphene oxide has functional groups that can be used to make many composite materials [18], which can be improved in terms of their mechanical, physical, biological, etc., characteristics when added to nanocomposites [19]. A study by Sun J [20] demonstrated that a dual coating of GO and silane exhibits improved anti-corrosion performance. Polarization tests via Tafel analysis showed that coatings with GO materials enhance anti-corrosion effects. One study [21] utilized the dispersibility of GO, using it as a carrier for SiC to fill coating defects, and the bonding between the materials provides possibilities for self-assembly. Adding GO can reduce cracks on the surface of HA coatings, increase corrosion resistance in SBF solutions, and enhance adhesion. Additionally, adding a small amount of GO can improve in vitro biocompatibility [22].

Zinc oxide nanoparticles (ZnONPs) are one of the most common nanometal oxide materials and belong to n-type semiconductors. They have a wide band gap of 3.37 eV and an excitation binding energy of 60 meV at room temperature [22]. They can be used in antibacterial drugs [23,24], anti-cancer treatment [25,26,27], and bone tissue engineering scaffolds [28], which can enhance biological activity, nucleic acid metabolism, and promote enzyme activity and biomineralization [29]. The release of zinc ions (Zn^2+^) and the generation of reactive oxygen species (ROS) grant ZnO bactericidal activity, which can confer antibacterial characteristics on the surface of the implant [30]. Studies have shown that zinc-doped hydroxyapatite nanoparticles (ZnO/HAps) have good biocompatibility and can enhance biological activity, promote nucleic acid metabolism, enhance enzyme activity, and promote biomineralization [29]. Therefore, this composite material can be used as a dental implant coating material for bone defect repair [31].

This study intends to use a simple and rapid hydrothermal treatment combined with microarc oxidation technology to load zinc oxide–graphene composite materials with a porous morphological surface structure onto the surface of Ti. By studying the physical and chemical properties of the surface morphology, elastic modulus, and wetting and corrosion resistance of the GO/ZnO/nHAp composite coating—as well as the biocompatibility of cell adhesion, proliferation, and mineralization, and the elastic modulus, corrosion resistance, and bone guidance of the GO/ZnO/nHAp composite coating—it provides a reference for further in vivo research and clinical applications.

## 2. Materials and Methods

### 2.1. Experimental Reagents

Table 1 shows the relevant information on the experimental reagents used in the preparation of the composite coatings, and Table 2 shows the relevant information on the reagents for the cell experiments.

### 2.2. Material Preparation

Nanometer calcium phosphate particles were prepared by the molecular template method—analytically pure CaCl_2_ solid and Na_2_HPO_4_·12H_2_O solid were selected as the calcium source and phosphorus source, respectively, and the solution was prepared according to a molar ratio of Ca to P of 10:6. Sodium EDTA-2Na was used as the template, the pH value of mixture was adjusted to 10, and the mixture was heated in an autoclave—the precipitate was naturally cooled to room temperature, and the product was collected by centrifugation and freeze-dried.

### 2.3. Sample Preparation

Titanium rods were cut into titanium sheets with a diameter of 15mm and a thickness of 2mm. The titanium sheets were then polished sequentially using different grit sizes of silicon carbide (SiC) sandpaper (600 #, 800 #, 1200 #, 1500 #, 2000 #), thoroughly polished, and ultrasonically cleaned with acetone, ethanol, and ultra-pure water for 15 min. After drying in an oven for 24 h, they were sealed and stored for later use. Micro-arc oxidation coating preparation: The electrolyte for this test was composed of KOH 5 g/L, KF 10 g/L, Na_2_SiO_3_ 6g/L, and NaPO_4_ 25 g/L. During preparation, add the reagents to distilled water in the above order while stirring. After all the reagents are added, stir thoroughly for 15 min. The temperature of the prepared microarc oxidation plating solution should not exceed 60 °C. The above specimens were placed at the anode of the micro-arc oxidation equipment, with graphite electrodes serving as the cathode, and a mixed aqueous solution used as the electrolyte. Each specimen was treated for 5 min at a frequency of 500 Hz, a pulse width of 50 μs, a voltage of 400 V, an ultrasonic frequency of 60 kHz, and an ultrasonic power of 50 W. After removing the specimens, they were rinsed with distilled water, air-dried naturally for 24 h, and then sealed and stored for later use. Hydrothermal method for preparing HA coatings: nHAp was stirred magnetically at 60 °C for 2 h. The milky white mixture was poured into a polytetrafluoroethylene inner pot, placed in a hydrothermal synthesis reactor, and heated to 160 °C for 24 h. After cooling the reactor, medical tweezers were used to remove the titanium sheets, which were then dried for 24 h and sealed to avoid light exposure for later use. GZH group coating preparation: Replace nHAp with GO/ZnO/nHAp composites containing different mass fractions (0.08%, 0.16%, and 0.32%) of GO/ZnO nanocomposites, with the rest being the same as the preparation method for the HA coatings.

### 2.4. Experimental Grouping

The specimens used in this study were divided into a control group (MAO) and four experimental groups: an HA group (HA), a GZH1 group (the GO/ZnO/nHAp group with 0.08 ω% GO/ZnO composite added), a GZH2 group (the GO/ZnO/nHAp group with 0.16 ω% GO/ZnO composite added), and a GZH3 group (the GO/ZnO/nHAp group with 0.32 ω% GO/ZnO composite added).

### 2.5. Characterization Methods

#### 2.5.1. Field Emission Scanning Electron Microscope (FE-SEM)

This experiment used an SU8010 field emission scanning electron microscope (FE-SEM, made by Hitachi, Tokyo, Japan) to first coat the specimen surface with gold. It then obtained information on the sample’s surface morphology and composition by detecting signals generated from the interaction between electrons and the sample.

#### 2.5.2. Energy Dispersive X-Ray Spectroscopy (EDS)

Energy dispersive spectroscopy (EDS) is a technique used for analyzing the chemical composition of materials. By detecting the characteristic X-rays emitted from a sample when it is excited by an electron beam, the types and concentrations of elements within the sample can be determined. This allows for the analysis of elemental distribution on coated surfaces.

#### 2.5.3. X-Ray Diffraction (XRD)

XRD uses X-rays interacting with crystalline materials to analyze their crystal structure and phase composition, thereby determining their crystal structure and phase composition. This test uses XRD (German BRUKER D8 ADVANCE, BRUKER AXS GMBH, Karlsruhe, Germany) under the following conditions: Cu-Kα radiation (voltage 36 kV, current 30 mA), within the 2 θ range of 20° to 80°, with a scanning speed of 2°/min.

#### 2.5.4. Raman Spectra (Raman)

Raman spectroscopy is an analytical technique based on Raman scattering, used to study molecular vibrations, rotations, and lattice modes. This experiment utilized the Witec Rlpha300 Raman spectrometer (Witec, Ulm, Germany) with a scanning wavelength of 532 nm.

#### 2.5.5. Atomic Force Microscopy (AFM)

AFM acquires high-resolution topographical and physical property information of a sample by detecting interatomic forces between the probe and the sample surface. In this study, roughness measurements were performed on dried specimens, with a test area of 10 μm × 10 μm selected.

#### 2.5.6. Fourier-Transform Infrared Spectroscopy (FTIR)

Sample preparation involved KBr pellet pressing, with the scanning spectrum recorded in the range of 400–4000 cm^−1^. The experiment was conducted using FTIR (German BRUKER VERTEX70, Karlsruhe, Germany) in transmission mode.

### 2.6. Physical and Chemical Property Test

#### 2.6.1. Coating Bond Strength

The coating bond strength is measured according to the standard GB/T 30707-2014 [32], which employs the principle of the load strength equivalent to the coating bond strength. The load strength is tested using the WS-2005 type automatic scratch tester for coatings developed by Lanzhou Zhongke Kehua Technology Development Co., Ltd. (Lanzhou, China). Test parameters: loading force 30 N, loading rate 30 N/min, scratch length 5 μm, constant load length 5 mm, number of scratch cycles: 1 (*n* = 3).

#### 2.6.2. Nanoindentation

The mechanical behavior of the composite coatings was evaluated using a nanoindenter (CPX-NHT2, AntonPaar, Switzerland). Load versus penetration depth curves were recorded under controlled constant conditions in the dynamic loading range—maximum load: 5.00 mN; loading rate: 10.00 mN/min; unloading rate: 10.00 mN/min pause: 5.0 s.

#### 2.6.3. Differential Thermal Analysis (TG-DSC)

The thermal stability of the materials was analyzed by a synchronous thermal analyzer (SDTQ600, TA Instruments Inc., Wakefield, MA, USA) in a nitrogen atmosphere with a heating range of 30 °C~1100 °C and a heating rate of 10 °C/min.

#### 2.6.4. Water Contact Angle

The water contact angle (n = 3) of the composite coating was measured by a (JC2000c, Zhongchen Digital, Shanghai, China) contact angle tester, and the average value was taken as the test result.

#### 2.6.5. In Vitro Mineralization Experiments

The ability to induce bone-like apatite formation was evaluated by immersing the sample in simulated body fluid (SBF) at 37 °C for 14 days. The SBF solution in centrifuge tubes was replaced every 48 h in a constant-temperature biochemical incubator set at 37 °C. The SBF composition was as follows: 8.036 g/L NaCl, 0.352 g/L NaHCO_3_, 0.225 g/L KCl, 0.230 g/L K_2_HPO_4_·3H_2_O, 0.311 g/L MgCl_2_·6H_2_O, 0.293 g/L CaCl_2_, and 0.072 g/L Na_2_SO_4_ dissolved in 1 L of deionized water. Finally, calibration was performed using 40 mL of 1.0 mol/L HCl solution and 3 g/L (CH_2_OH)_2_CNH_2_ solution at a pH of 7.4 and a temperature of 37 °C.

#### 2.6.6. Electrochemical Analyses

Using Tafel polarization (Temperature: 37 °C, pH = 7.4), the polarization curves of each group of coating samples (n = 3) were measured after immersion in the SBF solution for 10 min and 4 d, respectively. Before electrochemical testing, the open circuit potential (OCP) was established by immersing the samples in SBF for 60 min. The potential range of the potentiodynamic polarization test was −0.75~0.25 V, and the scan rate was 10 mV/s. The working electrode system used in traditional three-electrode batteries includes a saturated calomel electrode (SCE), a counter electrode (platinum), and a working electrode (coated substrate).

### 2.7. Cellular Experiments

#### 2.7.1. Cell Culture

After retrieving the frozen mouse pre-osteoblast cells (MC3T3-E1) provided by the College of Basic Medical Sciences at Jilin University, they were placed in a 37 °C water bath and agitated until thawed. They were then transferred into a sterile culture flask. The cells were cultured in a constant temperature incubator with 37 °C, 5% CO_2_, and 95% humidity. After 48 h, fresh DMEM medium was added, and the growth status of the cells was observed.

#### 2.7.2. DMEM Osteogenic Induction Medium

Dexamethasone 10 µg, vitamin C 25 mg, sodium β-glycerophosphate 1.08 g, and DMEM culture medium 500 mL.

#### 2.7.3. Cytotoxicity by CCK-8 Assay

MC3T3-E1 was inoculated into a 24-well plate at 3.0 × 10^4^/mL, cultured for 1, 3, 5, and 7 days, respectively, and the solution was changed every 2 days. In total, 10 μL of CCK-8 reagent was added into each well at each time point, and the OD value of each well was detected by a microplate reader at 450 nm. The cell survival rate (%) was calculated according to the absorbance value of each group, and the formula was as follows:

(1) $$\mathrm{Cell}\;\mathrm{survival}\;\mathrm{rate}\;(\%)=\frac{\left(ODexperimental\;group-ODblank\;control\;group\right)}{\left(ODnegative\;control\;group-ODblank\;control\;group\right)}$$

#### 2.7.4. ALP Activity Assay

The sample was placed in a 24-well cell culture plate and then a density of 1 × 10^4^ mL of MC3T3-E1 cells was seeded onto the sample surface. After 14 days of culture in a cell incubator, the cell culture medium was removed, and the samples were washed 3 times with disinfected PBS solution. In total, 200 μL of Triton-X100 was then added to a 24-well plate and kept overnight at 4 °C. The mixture was mixed with 30 μL of Triton-X100 and 100 mL of ALP (ALP, Activity Assay Kit, JC-A0059) as a detection solution, and this was then used as the absorbent detection solution at a potential of 520 nm. The total protein content in the 24-well plates was calculated using the BCA method. Finally, the ALP activity was calculated using the below formulas.

HOD = (COD − KOD)/(BOD − KOD) ZBCA/DBCA

In the formulas, HOD is the ALP activity, COD is the absorbance of the cell fluid containing Triton-X100 and ALP, KOD is the absorbance of the cell culture plates containing ALP, BOD is the absorbance of the cell culture plates, ZBCA is the standard concentration, and DBCA is the total protein mass in the cell culture plates.

#### 2.7.5. ARS Alizarin Red Assay

The deposition of calcium-containing compounds in the early stages of cell differentiation was evaluated by alizarin red S staining. A total of 1 × 10^4^ mL of MC3T3-E1 cells was inoculated on experimental specimens for 14 days, respectively. For evaluation, after 1 day of incubation, the medium was changed to osteogenic differentiation medium. The Alizarin Red S staining reagent was stained. After staining, 10% cetylpyridine chlorinated (CPC, Sigma, Shanghai, China) solution was added to the stained sample, and the absorbance was measured at 562 nm using a microplate photometer.

#### 2.7.6. Haemolysis Test

Fresh blood was collected from volunteers using vacuum blood collection tubes containing EDTA. The (Ti, MAO, HA, GZH1, GZH2, and GZH3) were placed in a 15 mL centrifuge tube, incubated in a constant-temperature water bath for 10 min, then 200 μL of blood diluent was added and incubated in the incubator for 60 min. A total of 200 pL of diluted blood was mixed with 5 mL of tridistilled water as a positive control, and 200 μL of diluted blood was mixed with 5 mL of 0.9% NaCI solution as a negative control. After incubation, the centrifuge tube was taken out, centrifuged at 1000 r/min for 10 min, and the absorbance of the supernatant was detected by a microplate reader at 545 nm. The hemolysis rate was calculated as follows:

(2) $$\mathrm{Hemolysis}\;\mathrm{rate}=\frac{\left(OD\;experimental\;group-OD\;negative\;control\right)}{\left(OD\;positive\;control-OD\;negative\;control\right)}\times 100\%$$

### 2.8. Statistical Analysis

Quantitative data were analyzed using SPSS22.0 (Chicago, IL, USA) and Origin2021 (Northampton, MA, USA). At least three experimental samples were collected for each test. The statistical methods used in this study include *t*-test and ANOVA. The mean ± SD was calculated, and *p* < 0.05 was considered statistically significant.

## 3. Results

### 3.1. Characterization of GO/ZnO/nHAp Composite Coating

#### 3.1.1. SEM of GO/ZnO/nHAp Composite Coating

**Figure 1:**
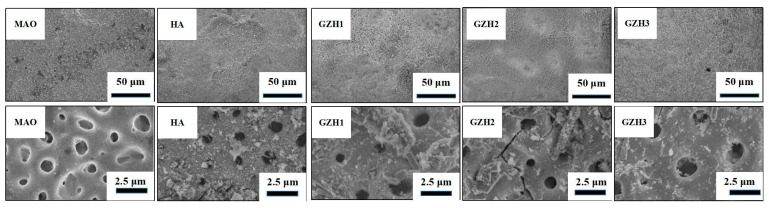
SEM observes the surface morphology of each group of coatings.

As shown in Figure 1, holes similar to a “crater” form (diameter < 2 μm) appeared on the surfaces of each group of coatings after microarc oxidation, with rough surfaces and flat structures. After hydrothermal treatment, the hydroxyapatite particles were scattered on the surface of the coating, and compared with the HA group coating—the hydroxyapatite particles on the GZH coating in the experimental group were denser, and a small number of rod-like structures could be observed.

#### 3.1.2. Energy Dispersion Spectrometer (EDS) Analysis

**Figure 2:**
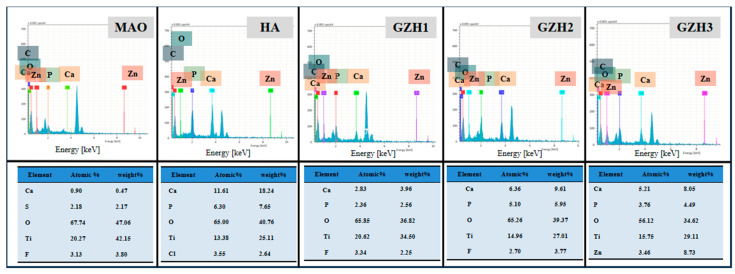
The content of surface elements in each group.

The EDS energy spectrum analysis of the composite coating is shown in Figure 2. It can be seen from the figure that each group has elements such as Ti, O, F, Ca, and P. This is because the electrolyte of MAO contains three elements—Ti, O, and F. After the hydrothermal treatment, the hydroxyapatite particles scattered on the surface of the coating contain Ca, P, and other elements. Compared with the HA group, the Ca and P element content in the GZH experimental group gradually increased. The Ca/P ratio of the GZH3 group was 1.66, which is close to the Ca/P ratio of natural bone tissue (1.67). At the same time, the element Zn appeared in the GZH3 group, indicating that the GO/ZnO/nHAp composite coating was successfully loaded onto the Ti surface, while no Zn was found in the GZH1 and GZH2 groups, which may be the reason for the low content of the GO/ZnO/nHAp composite materials.

#### 3.1.3. Surface Roughness

**Figure 3:**
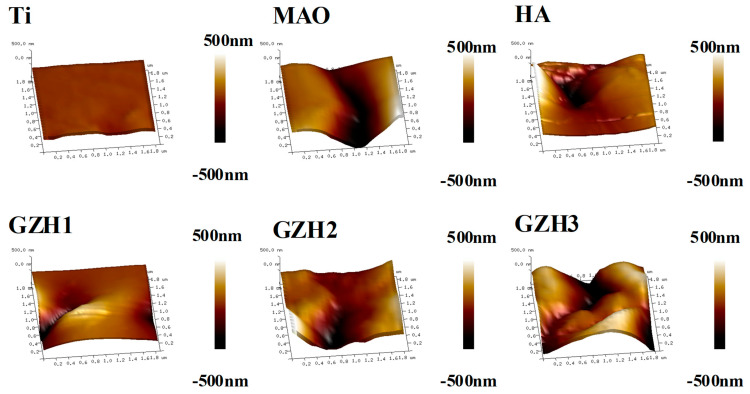
Three-dimensional morphology and surface roughness of each group by atomic force microscope.

The 3D morphology and surface roughness of each group of materials under atomic force microscopy (AFM) is shown in Figure 3. The surface roughness of each group of materials is Ti: Ra = 10.77 ± 1.91 nm; MAO: Ra = 163 ± 20.95 nm; HA: Ra = 108.57 nm ± 14.46 nm; GZH1: Ra = 89.30 nm ± 7.01 nm; GZH2: Ra = 129.00 nm ± 19.05 nm; and GZH3: Ra = 184.67 nm ± 12.74 nm. Among which, the surface roughness of the Ti group is the lowest, followed by the GZH1 group, and the GZH3 group has the highest roughness. The roughness of each group is as follows: Ti < GZH1 < HA < GZH2 < MAO < GZH3, because after microarc oxidation treatment, the “crater” form holes appear on the Ti surface, increasing the roughness of the Ti surface. When the GO/ZnO/nHAp content is small (as in the GZH1 and GZH2 groups), the coating is loaded onto the holes and the formation of relatively few hydroxyapatites will decrease the surface roughness; when the GO/ZnO/nHAp content is large (GZH3), a large number of nanorod-shaped or large nanosheet-shaped hydroxyapatite network structures is formed on the coating surface, increasing the surface roughness.

#### 3.1.4. FTIR Spectrum

**Figure 4:**
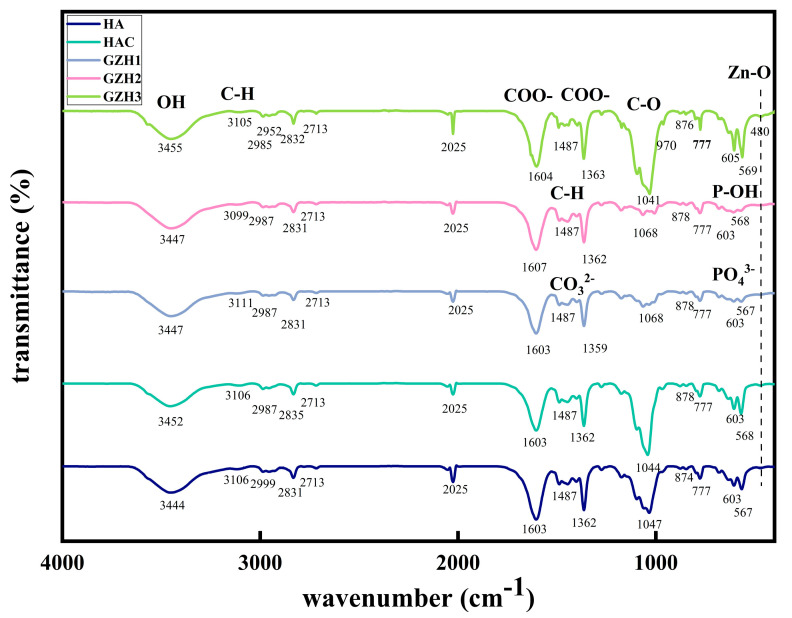
FTIR spectra of composite coatings.

The chemical bonds and functional groups in each group of materials were evaluated using FTIR spectroscopy. The results are shown in Figure 4. The absorption peak at 455 cm^−1^ is the stretching vibration of O-H; the absorption peak at 3105 cm^−1^ is the stretching vibration of unsaturated C-H; the absorption peak at 3000–2800 cm^−1^ is the stretching vibration of alkyl C-H; the absorption peaks at 1604 and 1363 cm^−1^ are the anti-symmetric and symmetric stretching vibrations of carboxylate COO-; the absorption peaks at 1604 and 1487 cm^−1^ are the skeleton vibration of the benzene ring; the absorption peaks at 1487 and 1399 cm^−1^ are the bending vibration of alkyl C-H; the absorption peak at 1447 cm^−1^ is the anti-symmetric stretching vibration of carbonate CO_3_^2−^; the absorption peaks at 1041 and 970 cm^−1^ are the anti-symmetric and symmetric stretching vibrations of PO_4_^2−^; the absorption peak at 876 cm^−1^ is the in-plane bending vibration of carbonate CO_3_^2−^; the absorption peak at 777 cm^−1^ is the out-of-plane bending vibration of the C-H benzene ring; the absorption peak at 605 cm^−1^ is the bending vibration of P-OH; and the absorption peak at 571 cm^−1^ is the anti-symmetric angular vibration of PO_4_^2−^. The peak at 480 cm^−1^ corresponds to the tensile vibration of the Zn−O−Zn and ZnO bonds. According to the analysis, the characteristic peaks of nHAp can be seen at PO_4_^3−^ (569 cm^−1^, 605 cm^−1^, and 970 cm^−1^) and -OH (3455 cm^−1^) in the HA group and the GZH composite coating group, and the peaks of ZnO can be seen at 480 cm^−1^, and the C-O peaks corresponding to GO at 1089 cm^−1^ and 1623 cm^−1^, respectively.

#### 3.1.5. XRD Test Results

**Figure 5:**
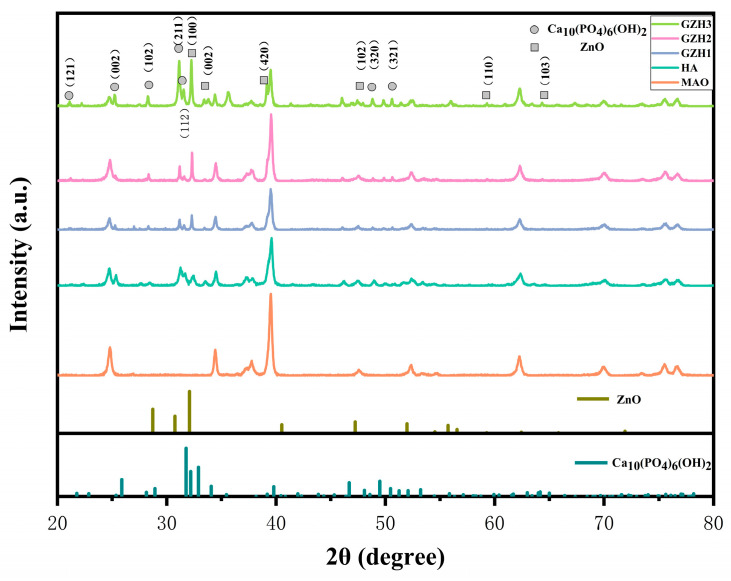
XRD spectra of composite coatings.

As shown in Figure 5, the XRD maps of each group of samples are shown in the sample group. Comparing with standard cards (Ca_10_(PO_4_)_6_(OH_2_), JCPDSNo.09-0432), in the XRD maps of four experimental groups, the diffraction peaks of nHAp can be observed at 2θ = 28.1°, 31.8°, 32.1°, 48.5°, and 50.7°, respectively, corresponding to the (102), (211), (112), (320), and (321) crystal planes in the HA phase. At 2θ = 32.4°, the sample’s crystal diffraction peak intensity gradually increased with the increase in GO/ZnO/nHAp content; at 2θ = 31.5 °, 34.2 °, and 46.6 °, respectively, it corresponded to the (100), (002), and (102) crystal planes in the ZnO (JCPDSNo.36-1451) phase. The above results show that the ZnO and HA in the HA and GO/ZnO/nHAp composites are successfully loaded onto the pure Ti surface after microarc oxidation. However, the GO phase was also not seen in the XRD spectra of the GZH experimental group, so we used Raman analysis to determine the presence of GO.

#### 3.1.6. Raman Spectra of GO/ZnO/nHAp Composite Coating

**Figure 6:**
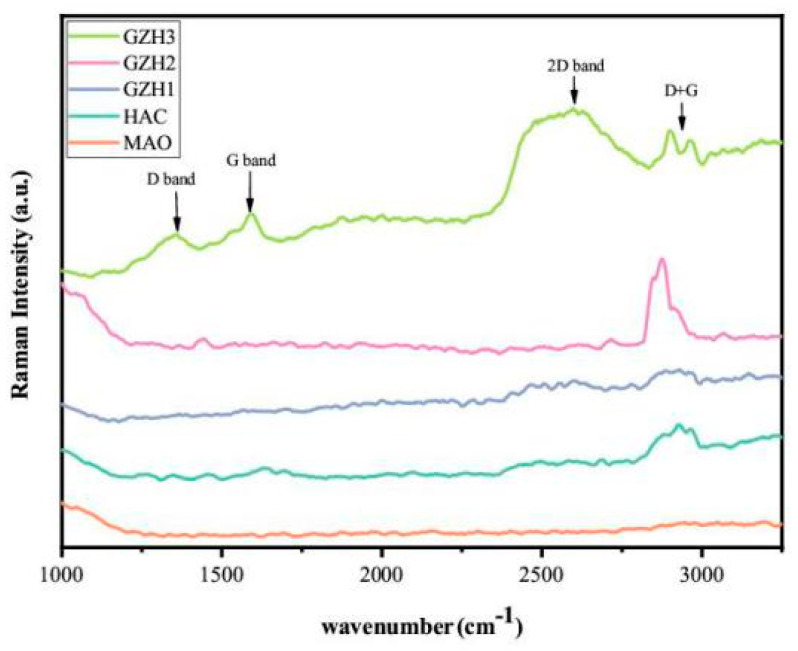
Raman diagram of composite coating.

The presence of GO in the experimental group GZH was verified by Raman spectroscopy. As shown in Figure 6, an obvious D-band (1355 cm^−1^) and G-band (1584 cm^−1^) appeared in the GZH3 group, indicating that GO has been successfully conjugated to the coating surface. Meanwhile, the GZH3 group is slightly offset compared with the D-band (1358 cm^−1^) and G-band (1590 cm^−1^) of pure GO, which may be caused by the phonon restriction generated by ZnO doping. However, the above two peaks did not appear in the experimental groups GZH1 and GZH2, which may be the reason for the low GO content of GO/ZnO/nHAp composites.

The characterization analysis of each group of composite coatings was performed by SEM, XRD, AFM, FTIR, EDS, and Raman spectroscopy. The results show that the GO/ZnO/nHAp composite was successfully loaded onto the pure Ti surface after microarc oxidation treatment.

### 3.2. Surface Wettability Detection Results

**Figure 7:**
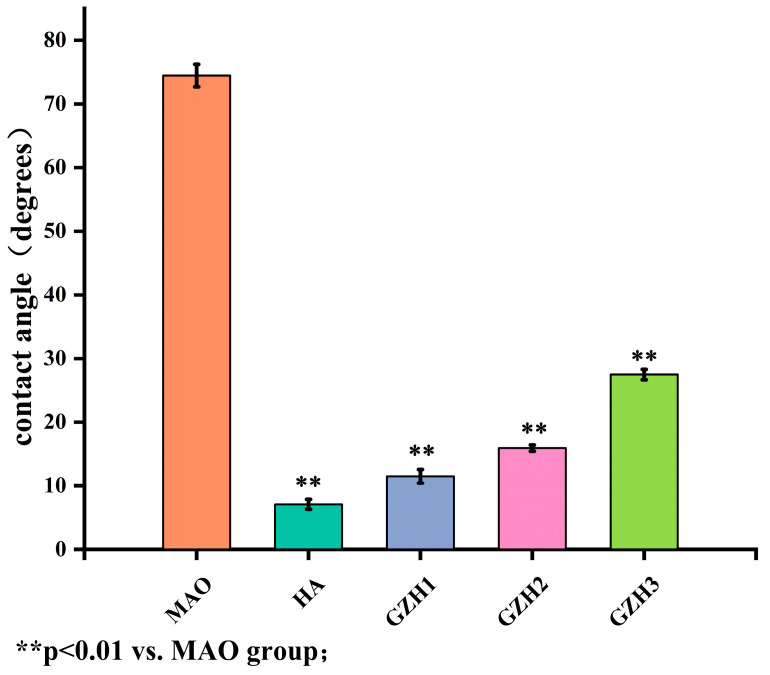
Test of water contact angles of sample surfaces of each group of composite coatings; ** *p* < 0.01 vs. MAO group.

The contact angle and surface free energy of the samples were imaged and calculated using a contact angle goniometer to evaluate the wettability of the samples. The results of each group were measured, as shown in Figure 7: The water contact angles of MAO, HA, GZH1, GZH2, and GZH3 were 74.5 ± 1.8°, 7.1 ± 0.8°, 11.5 ± 1.1°, 16.0 ± 0.5°, and 27.4 ± 0.8°, respectively. Compared with the MAO group, the water contact angles of each group in the experimental group were statistically significant (*p* < 0.05), and all showed good hydrophilicity, indicating that the pure TI surface loaded with the composite coating was a hydrophilic surface, which makes it conducive to early adhesion, added value, and the differentiation of cells.

### 3.3. Differential Thermal Analysis (TG-DSC)

**Figure 8:**
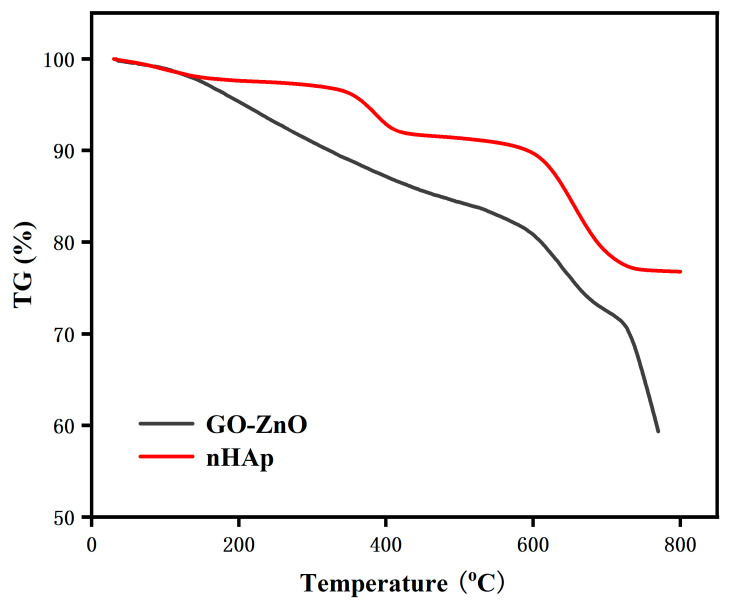
Composite coating TG diagram.

**Figure 9:**
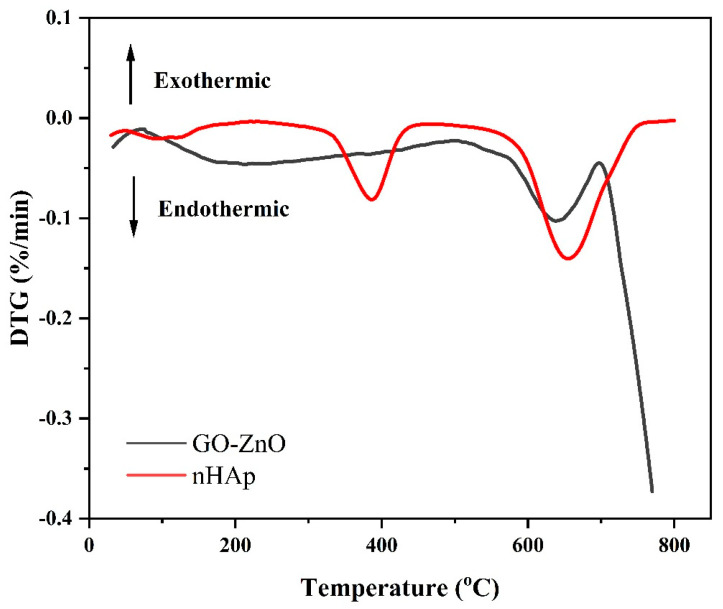
Composite coating DSC diagram.

The TG and DSC curves of the composite coatings are shown in Figure 8 and Figure 9. From the figures, we can see that the thermal decomposition process of nHAp is roughly divided into three stages. The initial stage is within the range of 25~260 °C, and only a 3% mass loss occurs at this time; the second stage is between 260~500 °C, and the mass loss increases to 6%; the third stage is between 500~800 °C, and the mass loss significantly increases to 14%; the total mass loss of nHAp across these three stages is 23%.

The thermal decomposition process of GO-ZnO can also be divided into three stages. The first stage is from 25 °C to 530 °C, where the mass loss reaches 17%, which may be caused by the volatility of moisture on the surface of the material; the second stage is from 530 °C to 710 °C, and the mass loss here is 12%, which may be caused by the loss of water present in the GO; entering the third stage, that is, within the range of 710~800 °C, the mass loss is again 12%, which may be related to the formation of CO, CO_2_, and water vapor by pyrolyzing the oxygen-containing functional groups; the total mass loss of GO-ZnO across these three stages is 41%.

Overall, nHAp exhibits higher thermal stability compared with GO-ZnO. This is not only reflected in the temperature threshold of the rapid mass loss, but the final mass retention is also fully demonstrated. The excellent thermal stability of nHAp is closely related to the phosphorus it contains. The presence of phosphorus can significantly improve the thermal stability of a material, thereby maintaining its structure and performance stability in high temperature environments [33].

### 3.4. Adhesion Strength of the Coating

**Figure 10:**
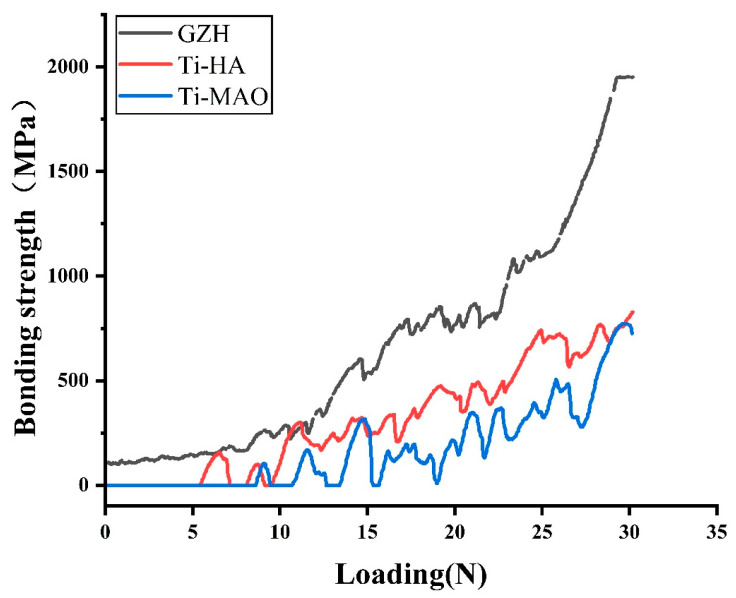
Coating adhesion strength.

The adhesion of the coating to the substrate was tested using a WS-2000 automatic scratch tester. Three samples were randomly selected for testing, with three measurements taken from different areas of each sample, and the average value was calculated. The results are shown in Figure 10. The critical load for Ti-MAO is 8.8 ± 4.2 N, for Ti-HA it is 4.9 ± 1.7 N, and for Ti-GZH it is 10.1 ± 3.4 N. The *t*-test results indicate a significant difference between the Ti-HA group and the Ti-MAO and Ti-GZH groups (*p* < 0.05).

### 3.5. Nanoindentation

**Figure 11:**
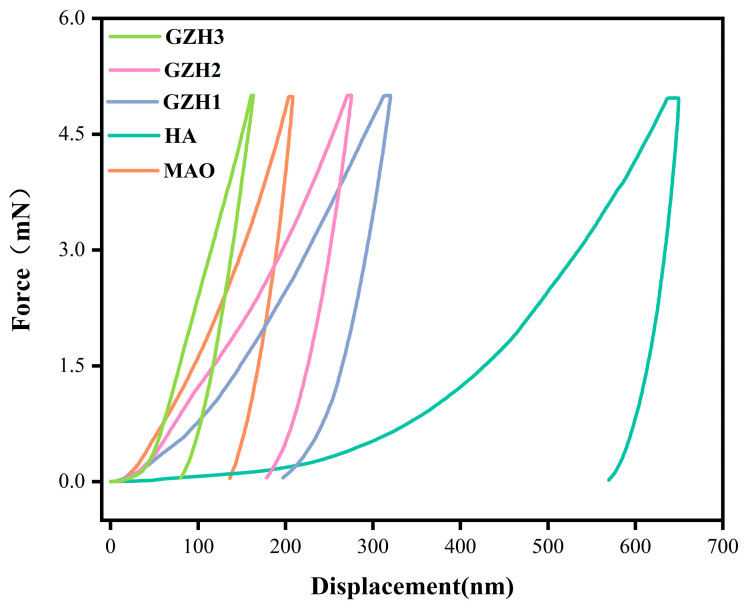
Nanoindentation Test on Surfaces of Samples in Each Group of Composite Coatings.

The mechanical properties of each group of coatings were evaluated by the nanoindentation method, and the results are shown in Figure 11 and Table 3. The penetration depth of the GZH groups of coatings was lower than that of the HA group of coating, which indicates that the mechanical properties of the GZH groups of coatings were higher than that of the HA coatings. Table 1 shows the nanoindentation test data of each group of coatings under a 5 mn load. From the data in the table, it can be seen that the nanohardness (H), Young’s modulus (E), and H/E of the GZH experimental group were higher than those of the HA group, indicating that the GZH coating has a stronger ability to withstand critical loads from the elastic mode to the plastic mode. The mechanical properties of metal substrate coatings are key parameters that must be considered. Poor mechanical properties of a coating will cause various types of corrosion to the substrate, which will lead to damage to the substrate in the long run. The loaded GO/ZnO/nHAp composite coating improves the mechanical properties of Ti in a way that may be due to the synergistic action of ZnO and GO. The excellent mechanical properties of the two enhance the bending strength and fracture toughness [34]. The *t*-test results indicate no significant difference in elastic modulus (E) between the MAO group and the GZH3 group (*p* > 0.05). All other groups showed significant differences compared to the Ti-MAO group (*p* < 0.05).

### 3.6. In Vitro Mineralization Experiments

**Figure 12:**
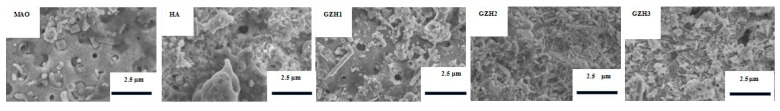
SEM images of in vitro mineralization of composite coating samples from different groups.

**Figure 13:**
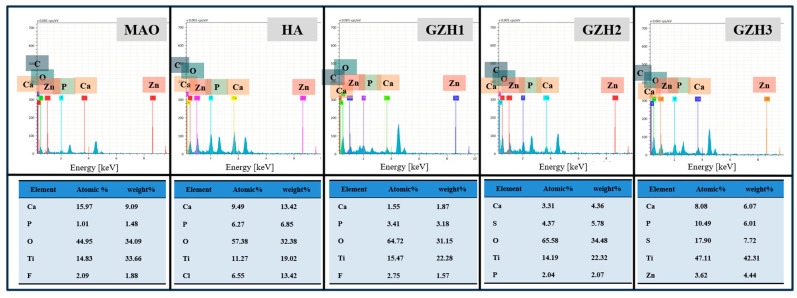
EDS images of in vitro mineralization of composite coating samples from each group.

The SEM images of each group of specimens after soaking in SBF solution for 14 days are shown in Figure 12. As shown in the figure, it was observed that the MAO group formed a small amount of white granular hydroxyapatite. Compared with the MAO group, the HA group was covered with a new layer of hydroxyapatite in the presence of a coating and agglomeration occurred. The degree of growth of hydroxyapatite in each GZH group was higher than that in the MAO group, and with the increase in GO/ZnO/nHAp content, the apatite covering on the surface increased. The EDS images in Figure 13 show that, after immersion in SBF, the characteristic elements Ca, P, and O, necessary for the formation of a hydroxyapatite layer, can be detected on each surface, with an increase in the intensity of the Ca and P peaks in the coated samples.

### 3.7. Electrochemical Test

**Figure 14:**
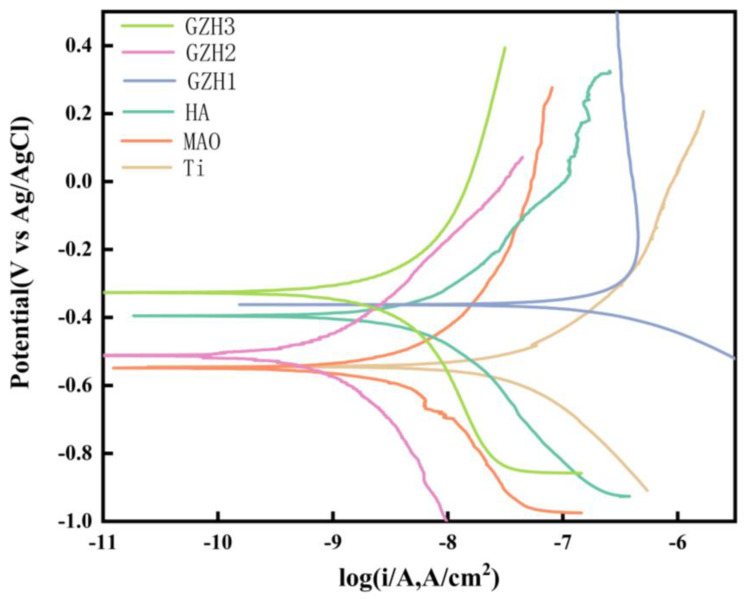
Tafel curves of each group of samples after soaking in SBF for 10 min.

The electrochemical corrosion behavior of the surfaces of each group of samples was studied by dynamic polarization test. It can be seen from Figure 14 and Table 4 that, after soaking in SBF solution for 10 min, the sample Icorr of the MAO group was 1.17 ± 1.60 × 10^−7^ A·cm^−2^ lower than the pure Ti group at 3.54 ± 1.89 × 10^−7^ A·cm^−2^, while the sample Icorr of the GZH2 group was 1.94 ± 3.29 × 10^−7^ A·cm^−2^, which was lower than the HA group at 2.57 ± 4.00 × 10^−7^. All samples were lower than the HA group at −0.34 ± 0.076 V, indicating that microarc oxidation treatment can in the short term increase the corrosion resistance of the pure Ti surface. The Icorr and Ecorr in the experimental groups (GZH1, GZH2, and GZH3) both showed first a decrease and then an increase. The Icorr and Ecorr in the GZH2 group were similar to the HA group, indicating that the addition of GO/ZnO/nHAp composites failed to show significant advantages in the short term (*p* > 0.05).

**Figure 15:**
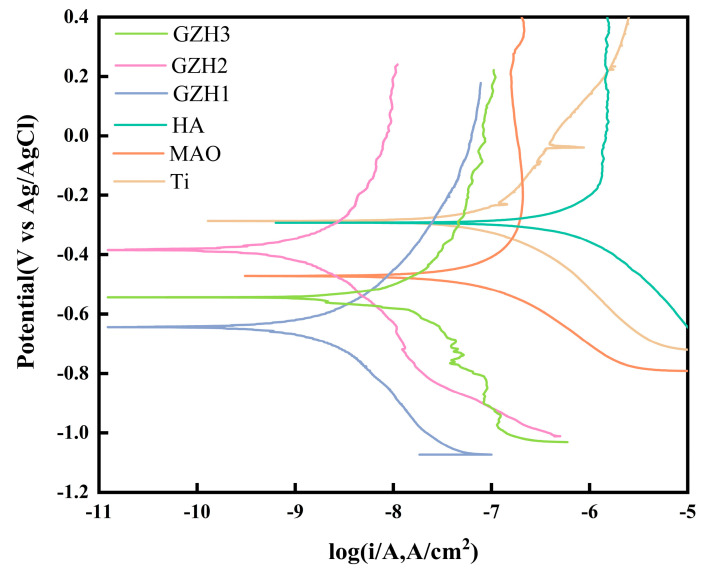
Tafel curves of each group of samples after soaking in SBF for 4 days.

As shown in Figure 15 and Table 5, after 4 days of SBF solution immersion, the Icorr value of the GZH sample was lower than that of the MAO group and the HA group. Among the groups, the GZH2 group (0.99 ± 1.39 × 10^−8^ A/cm^2^) had a lower current density compared to the GZH1 group (1.01 ± 1.66 × 10^−7^ A/cm^2^) and the GZH3 group (6.66 ± 4.70 × 10^−8^ A/cm^2^), and each composite coating group showed significant differences compared to the HA group (*p* < 0.05). The order of corrosion resistance from best to worst is GZH2 > GZH3 > HA > GZH1 > MAO > Ti. The results indicate that the corrosion resistance of coatings containing GO/ZnO/nHAp composite materials gradually increases over time.

Therefore, a proper amount of GO/ZnO/nHAp composite coating (GZH2) can improve the corrosion resistance of titanium surface, which may be related to the fact that GO contains oxygen-containing functional groups, which can effectively reduce the migration rate of electrons to GO; that is, GO acts as a barrier in the process of electron migration, thus improving the corrosion resistance of the titanium substrate [35].

### 3.8. MC3T3-E1 Cell Cytological Testing Results

#### 3.8.1. CCK-8 Detection Results of MC3T3-E1 Cell Proliferation on the Surface of Each Group of Samples

**Figure 16:**
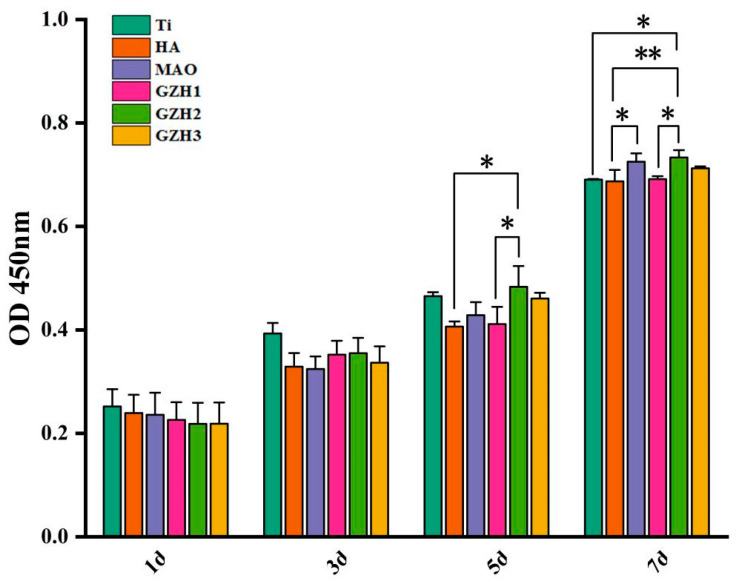
CCK-8 of composite coatings; * *p* < 0.05, ** *p* < 0.01.

The CCK-8 detection results of MC3T3-E1 cell proliferation on the surface of samples in each group are shown in Figure 16. After all the samples were co-cultured with MC3T3-E1 cells, the cells grew well. The cell proliferation in the 1st and 3rd days was not statistically significant (*p* > 0.05), while the cell proliferation on the 5th and 7th days in the GZH2 group was statistically significant (*p* < 0.05), which indicated that the samples in each group had good biocompatibility and promoted cell adhesion and proliferation.

**Figure 17:**
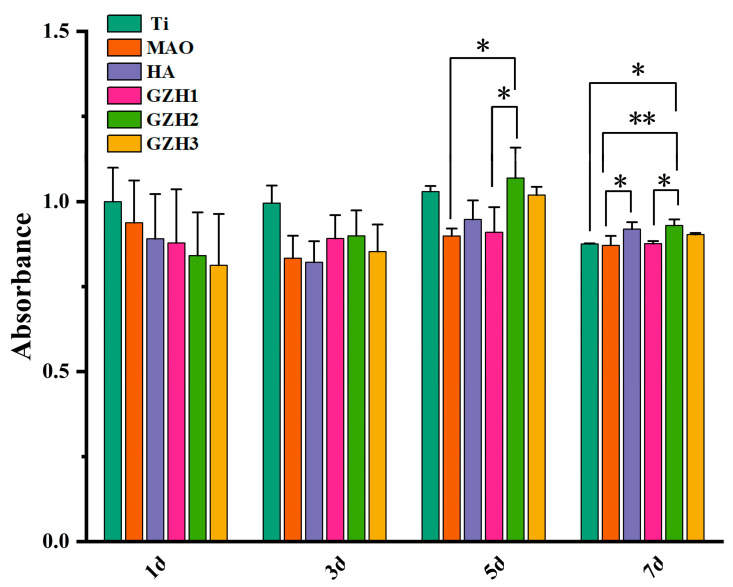
Cell absorbance of composite coatings; * *p* < 0.05, ** *p* < 0.01.

Figure 17 shows the relative cell proliferation rate of each group of specimens after co-culture with MC3T3-E1 cells. Compared with the MAO group at the same time after co-culture, the MC3T3-E1 cells in the four experimental groups grew and reproduced well without obvious cytotoxicity. There was no significant difference between 1 d and 3 d. On the 5th and 7th days, cell proliferation in GZH2 was statistically significant compared with the MAO group. According to the ISO10993-5 standard (Table 6), the cytotoxicity of the six groups of specimens is 0~1, which indicates that the GZH coating has good biocompatibility [36].

#### 3.8.2. Alkaline Phosphatase (ALP)

**Figure 18:**
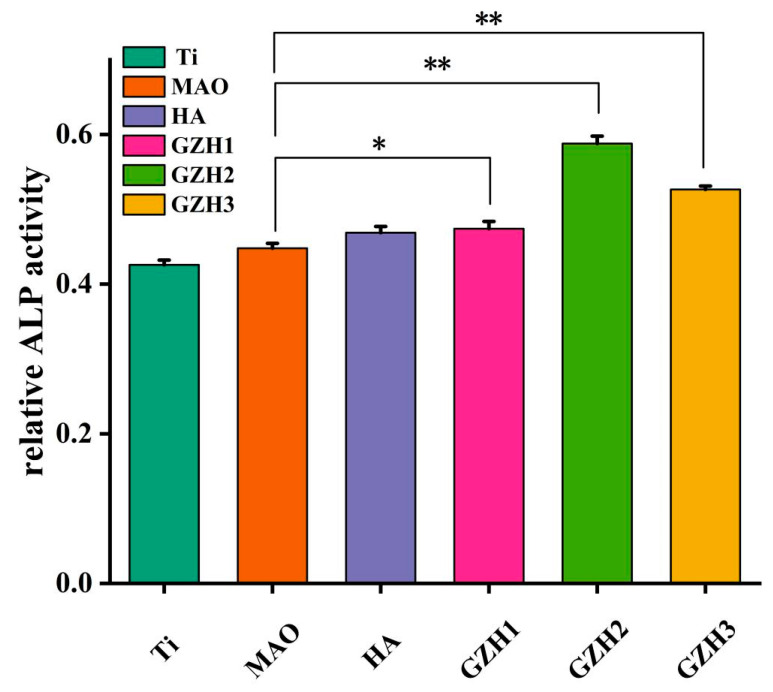
ALP of composite coatings; * *p* < 0.05, ** *p* < 0.01.

After 7 days of co-culture, the ALP expression of MC3T3-E1 cells was analyzed to evaluate early osteogenic ability. As shown in Figure 18, the expression of ALP was GZH2 > GZH3 > GZH1 > HA > MAO > Ti, and compared with the MAO group, at the same time, the three groups GZH1, GZH2, and GZH3 were all statistically significant. It is shown that a GO/ZnO/nHAp composite coating can promote the directional differentiation of MC3T3-E1 cells to osteoblasts and promote early osteogenesis.

#### 3.8.3. Alizarin Red (ARS)

**Figure 19:**
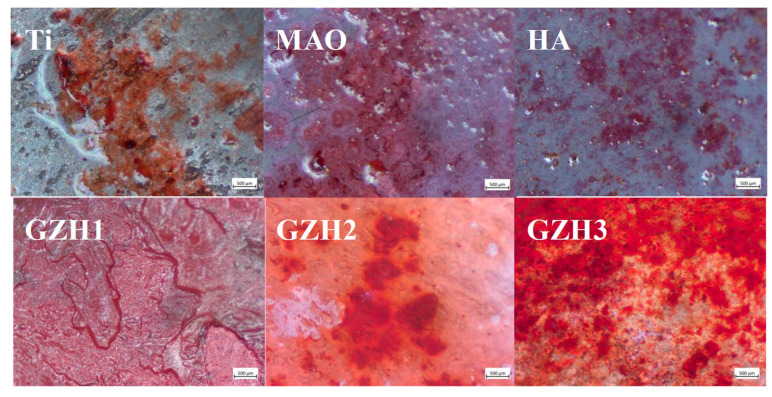
Microscopic images of the composite coatings’ ARS chromosomes.

**Figure 20:**
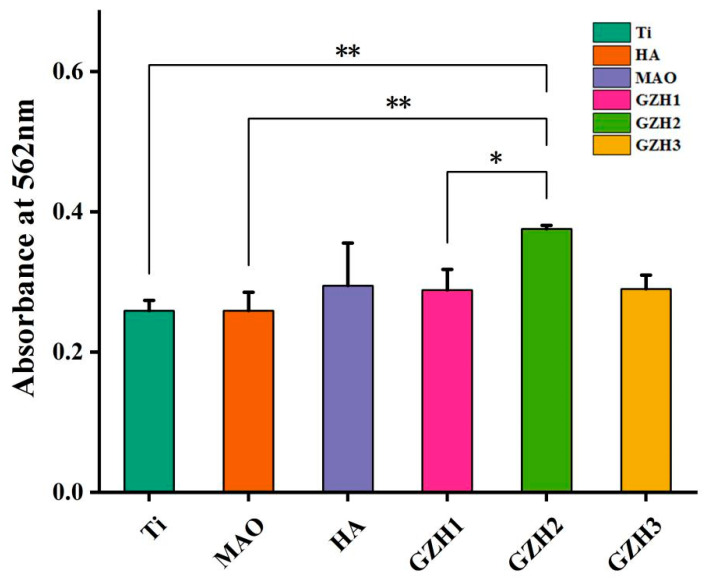
Bar chart of quantitative analysis of composite coatings’ ARS staining; * *p* < 0.05, ** *p* < 0.01.

The asana microscope image of alizarin red stained after co-culturing the specimen with MC3T3-E1 cells for 14 days is shown in Figure 19. It can be seen that the purple-orange-red coloring is mineralized nodules. Among the groups, the GZH2 group has the most orange mineralized nodules and the strongest mineralization ability. It is followed by the GZH3 group, the HA group, and the GZH1 group. The Ti-group and MAO group have a relatively light coloring, fewer mineralized nodules, and weak mineralization ability. In the quantitative analysis of the ARS in Figure 20, the GZH2 group exhibited the highest degree of mineralization, which was statistically significant compared to the MAO group at the same time (*p* < 0.05), and is consistent with the results of the Alizarin Red S staining.

#### 3.8.4. Hemolysis Experiment

**Figure 21:**
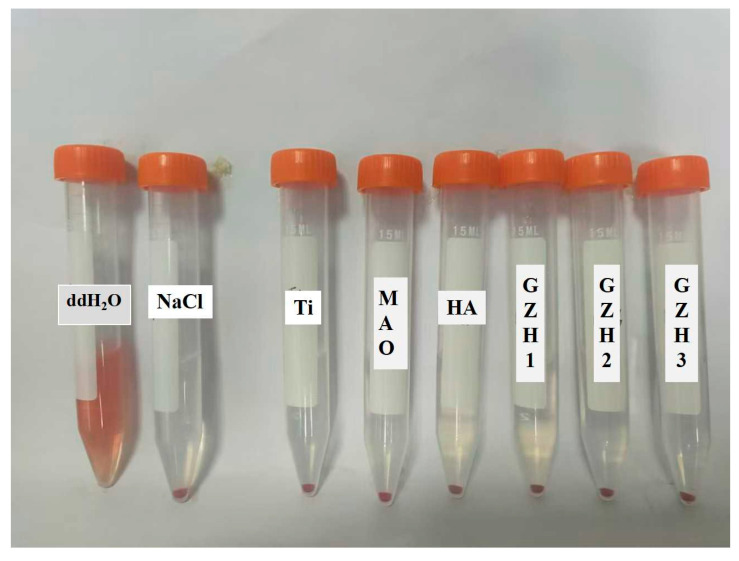
Effect of mixing after centrifugation.

**Figure 22:**
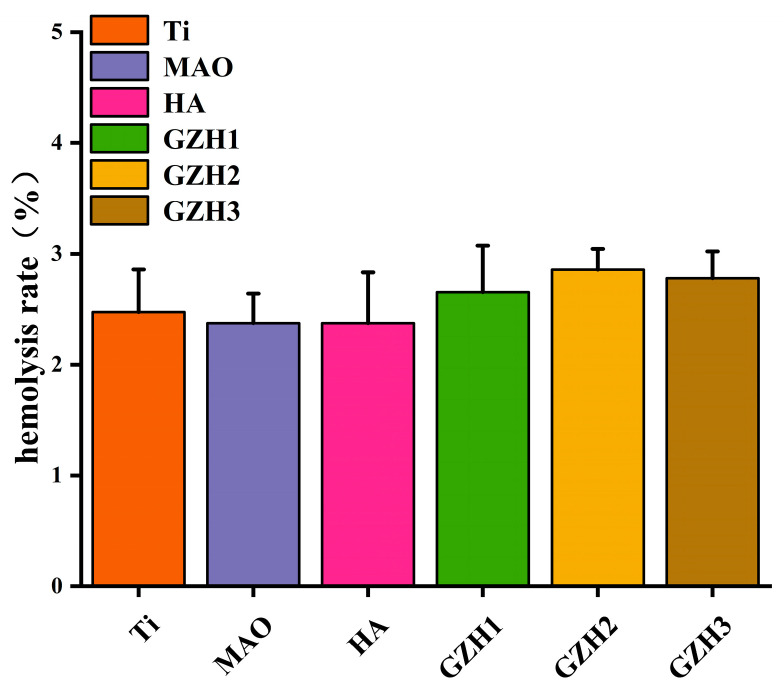
Hemolysis rate bar chart.

As shown in Figure 21, both the experimental group and the negative control group exhibited layering after centrifugation. The upper layer consisted of a clear liquid, while the lower layer contained red precipitate, which was the deposited red blood cells. Hemolysis occurred in the positive control group. The OD values of the supernatants were measured, and the hemolysis rates were calculated, with results being presented in Figure 22. The hemolysis rates for all groups were less than 5%, meeting the requirements for relevant standard hemolysis tests. This indicates that the composite coatings are non-toxic or are minimally toxic to red blood cells, do not induce hemolysis reactions, and are safe for and feasible in clinical applications.

## 4. Discussion

Titanium and its alloys are widely used in clinical practice [37], but there is still a risk of transplant failure [38]. Next-generation implants need to have good corrosion resistance [4], biocompatibility [5], and antibacterial ability [39] to have scientific and clinical value. Previous studies have used the method of combining MAO and HT to modify pure Ti, and have successfully obtained nanoscale surfaces and high hydrophilicity [40]. Hydroxyapatite, as a natural polymer, is often selected as an implant coating material, which helps improve biocompatibility and optimize osteobinding capabilities [41]. Zinc oxide has antibacterial activity and also exhibits antioxidant and anticancer cell properties [42]. Graphene can be used to improve the mechanical properties of materials [6].

A TiO_2_ ceramic substrate is formed through the MAO process, and TiO_2_ is used as the intermediate layer. Then, the GO/ZnO/nHAp composite coating is prepared by the hydrothermal method to give full play to the synergistic effect between materials, improve the inertia of the titanium surface, enhance biocompatibility, reduce the elastic modulus to fit human bones, improve corrosion resistance, and thus extend the service life of the implant. In this study, a GO/ZnO/nHAp composite coating was constructed on the Ti surface through MAO and HT, which significantly enhanced the mechanical properties including hydrophilicity, elastic modulus, etc., effectively improving the corrosion resistance and promoting the adhesion, proliferation, and differentiation of MC3T3-E1 cells, which was of positive significance for improving the osteogenesis ability and enhancing the effect of osteointegration.

However, biomaterial infection caused by biofilms on the surface of an implant is a major clinical problem [43]. It is particularly necessary to use microarc oxidation (MAO) technology to reduce the risk of infection and promote osteocyte response [40]. Microarc oxidation is an electrochemical surface modification technology [37]. By applying a high voltage between a metal and electrode, a ceramic oxide film is generated on a metal substrate [38]. This ceramic coating has the characteristics of a strong binding force and a dense and porous structure, which helps to control the release of metal ions [44]. Compared with plasma spraying technology, MAO has the advantages of lower cost, being easy to control, and requiring a lower temperature, which can effectively solve the problems of plasma spraying causing the decomposition of HA coatings at high temperatures [42,45], and the coating peeling off the surface of the implant and causing the implant to fail [41]. However, the pores formed by microdischarge during the MAO process may have a negative impact on corrosion resistance. There have been research attempts to combine MAO with other coating technologies to improve surface performance. Kossenko et al. successfully prepared a hydroxyapatite coating on a Ti-6AL-4V substrate by combining MAO and hydrothermal treatment [43]. This study aimed to load GO/ZnO/nHAp coatings with different proportions of GO/ZnO/nHAp after MAO treatment. Through fine adjustment of the elastic modulus and hardness, a suitable proportion of GO/ZnO/nHAp coating with a higher hardness that is closer to the elastic modulus of human bones was able to significantly improve the mechanical properties of the implant.

Software analysis of AFM images revealed that the roughness of the MAO group was higher than that of the pure TI group, indicating that the microarc oxidation treatment can increase surface roughness [45]. As shown in Figure 3, compared with the MAO group, the roughness increased after adding GO/ZnO. The inhomogeneity of the microarc oxidation discharge and the adhesion of composite materials lead to an increase in roughness. It can be observed from the SEM images in Figure 1 that a porous ceramic layer was formed on the coating surface, and spherical particles and nanorod-like structures were found on the MAO surface. Studies have shown that the increase in surface roughness is conducive to the proliferation and differentiation of osteoblasts [46]. EDS detection showed that the content of Ca and P elements in the MAO group was lower than that of the other coating groups, which indicated that the hydroxyapatite material successfully adhered to the pure Ti surface, which was consistent with the research results of JiMK et al. [47]. In addition, F is present in each group of coatings, which is related to the KF contained in the electrolyte. The Ca/P ratio of the coatings of the GZH groups was 1.66, which is close to the Ca/P ratio of natural bone tissue. The characteristic G and D peaks of graphene oxide appeared in the Raman spectrum, confirming the presence of graphene oxide on the surface of the composite coating. Compared with the G band of 1590 cm^−1^ and the disordered band of 1358 cm^−1^ (D band) of pure graphene oxide, there is a difference due to the phonon restriction caused by defects caused by ZnO doping. It may be that, after zinc oxide doping, the disorder of the graphite increases, making the G band wider and the relative strength of the D band increase [48]. The surface wettability test results show that the composite coating forms a hydrophilic surface. Some studies have pointed out that hydrophilic surfaces are conducive to promoting the adhesion and proliferation of osteoblasts [49]. The water contact angle measurements indicate that the composite coating formed a hydrophilic surface. Research has shown that hydrophilic surfaces are conducive to promoting osteoblast adhesion and proliferation [47]. In this experiment, the hydroxyapatite coating exhibited the best wettability, while the wettability of the composite coating group was lower compared to the hydroxyapatite coating, which may be related to the hydrophobic effect of the graphene in the graphene oxide–zinc oxide composite material [48]. The differential thermal analysis results indicate that HAp exhibits only a 3% mass loss between 25 °C and 260 °C, suggesting that nHAp remains stable in this temperature range with no significant decomposition. GO-ZnO shows a 17% mass loss between 25 °C and 530 °C, with no signs of intense decomposition, indicating that the composite coating is relatively stable and does not undergo noticeable pyrolysis at 121 °C, thus meeting the standard sterilization requirements for use as a biomaterial.

The mechanical properties of metal substrate coatings are key parameters that need to be considered in coating applications. If the coating has poor mechanical properties, it will cause various forms of corrosion to the substrate, which will cause damage to the substrate in the long term. Hardness and elastic modulus are two important characteristics that determine the mechanical properties of a coating. The elastic modulus of a coating should be similar to the elastic modulus of natural bone, and the hardness should be high enough to withstand induced mechanical loads after implantation. As shown in Table 1, the elastic modulus of the MAO sample was higher than that of natural bone in this experiment, and the HA coating was closer to natural bone than the substrate. The elastic modulus of the GZH1 and GZH2 groups increased compared with the HA group, but it was still close to the elastic modulus of human cortical bone (30 Gpa). Compared with the HA group, the hardness E of the coating groups increased with increases in the proportions of GO/ZnO/nHAp composite. The results of the GZH groups show higher H/E ratios. By assessing the elastic behavior of the coatings, the higher H/E ratios indicate that the GZH coatings had a stronger ability to withstand critical loads from the elastic mode to the plastic mode [50]. This experiment involved loading a composite coating of GO/ZnO/nHAp onto a titanium substrate after microarc oxidation pretreatment. The elastic modulus of this coating is significantly lower than that of pure titanium (approximately 115 GPa) and is more closely aligned with normal bone tissue (10–30 GPa) [51]. Although the coating, as a surface functional layer, cannot alter the overall elastic modulus of the implant, the low modulus interfacial layer effectively reduces the mismatch in elastic modulus between the titanium substrate and bone tissue, optimizes stress transfer pathways, and minimizes stress concentration in the interface region. This helps mitigate the stress shielding effect caused by the high modulus of traditional titanium implants [52,53]. The electrochemical polarization curve results show that the corrosion resistance performance brought about by soaking the coating group samples for 10 min in SBF solution is not obvious. After soaking the samples in SBF solution for 4 days, the corrosion current density of the GO-ZnO composite experimental group was lower than that of the HA group, which shows that the addition of the GO-ZnO composite can reduce the corrosion tendency compared with the HA coating. WangQ’s [54] research shows GO’s corrosion resistance, and that adding GO to composite materials can improve their corrosion resistance. In this experiment, the CCK-8 method was used to evaluate the activity of biological cells. The experimental results show that all of the coatings showed increased cell proliferation from day 1 to day 7. After 5 or 7 days of MC3T3 cell culture, the proliferation capacity of cells on the GO/ZnO/nHAp coatings was higher than that on the microarc oxidation coatings. On day 7, there were statistical differences between the HA group and the MAO group, which indicated that the addition of HA improved the biocompatibility of the MAO surface and enhanced the cell proliferation ability [55]. The alkaline phosphatase activity showed that the ALP activity of each GZH group was higher than that of the MAO group, indicating that the GO/ZnO/nHAp composites were capable of early osteogenesis. WuS’s [56] study also showed that HA has the effect of promoting the improvement of ALP vitality. Alizarin Red S staining and its quantification showed that the average mineralization value of the GO/ZnO/nHAp composite coating was higher than that of the MAO group, indicating that GO/ZnO can enhance the expression of osteogenic differentiation markers and promote calcium deposition. The hemolysis rate can reflect the strength of the interaction between the material and the red blood cells after contact. If the hemolysis rate of the material is low, this indicates that the material causes less damage to blood cells (mainly red blood cells) and has better compatibility. The ISO/TR7405-1984 standard [57] stipulates that when the hemolysis rate is less than 5%, this indicates that the material will not cause a hemolysis reaction. The hemolysis rate of all the experimental groups in this experiment was less than the 5% specified in the standard, indicating that the composite coating prepared by the hydrothermal method after microarc oxidation treatment does not cause hemolysis reactions, and will not lead to hemolysis in clinical practice.

## 5. Conclusions

The GO/ZnO/nHAp composite coating was successfully constructed onto the surface of pure titanium after microarc oxidation. It improved the biological activity of the titanium surface, increased its hydrophilicity, improved its corrosion resistance, and reduced its elastic modulus so that the elastic modulus of the coating surface was more closely matched to the elastic modulus of normal human bone tissue. This study provides relevant experimental reference data for further study of the surface modification of pure titanium materials, and has certain application value and prospects in the treatment of bone defects caused by inflammation and tumors in their later stages.

## Figures and Tables

**Table 1:** Specifications and Manufacturers.

Chemical Formula	Grade	Manufacturer
CaCl_2_	Analytical reagent	Sinopharm Chemical Reagent Co., Ltd. (Shanghai, China)
Na_2_HPO_4_	Analytical reagent	Sinopharm Chemical Reagent Co., Ltd.
C_10_H_14_N_2_Na_2_O_8_	Analytical reagent	Macklin (Shanghai, China)
CH_3_COCH_3_	Analytical reagent	Sinopharm Chemical Reagent Co., Ltd.
C_2_H_6_O	Analytical reagent	Sinopharm Chemical Reagent Co., Ltd.
NaOH	Analytical reagent	Tianjin Kaitong Chemical Reagent Co., Ltd. (Tianjin, China)
KOH	Analytical reagent	Tianjin Kaitong Chemical Reagent Co., Ltd.
KF	Analytical reagent	Tianjin Kemio Chemical Reagent Co., Ltd. (Tianjin, China)
Na_3_PO_4_·12H_2_O	Analytical reagent	Tianjin Ruijinte Chemical Pharmaceutical Co., Ltd. (Tianjin, China)
Na_2_SO_3_·9H_2_O	Analytical reagent	Tianjin Hengxing Chemical Reagent Manufacturing Co., Ltd. (Tianjin, China)
GO/ZnO		Provided by Professor Lu Yuguang’s team of the School of Pharmacy, Jiamusi University (Jiamusi, China)

**Table 2:** Cells, kits, and manufacturers.

Name	Manufacturer
MC3T3-E1 osteoblasts	Provided by Basic Medical College of Jiamusi University (Jiamusi, China)
Alkaline Phosphatase Assay Kit	Beyotime Biotechnology (Shanghai, China)
BCA Protein Assay Kit	Beyotime Biotechnology
TritionX-100	Beyotime Biotechnology
Fetal Bovine Serum	Gibco company (Shanghai, China)
DMEM Medium with High Glucose	Hyclone company (Hyclone, NY, USA)
PBS	Hyclone company (Paisley, UK)
Trypsin-EDTA Soiution	Gibco company
4% Paraformaldehyde Fix Solution	Solomen company (Shanghai, China)
Cell Counting Kit-8	Beyotime Biotechnology

**Table 3:** Elastic modulus and Young’s hardness of each group of samples of composite coatings.

Parameter	MAO	HA	GZH1	GZH2	GZH3
H (GPa)	3.6 ± 1.1	0.4 ± 0.1	1.7 ± 0.1	2.7 ± 1.0	5.6 ± 1.0
E (GPa)	93.5 ± 6.0	40.8 ± 2.4	44.3 ± 3.1	53.5 ± 1.0	89.6 ± 3.4
H/E	0.039	0.010	0.038	0.050	0.63

**Table 4:** Corrosion current density and corrosion voltage in each group of samples after soaking in SBF for 10 min.

Samples	Icorr (A/cm^2^)	Ecorr (V vs. Ag/AgCl)
Ti	3.54 ± 1.89 × 10^−7^	−0.46 ± 0.081
MAO	1.17 ± 1.60 × 10^−7^	−0.62 ± 0.125
HA	2.57 ± 4.00 × 10^−7^	−0.34 ± 0.076
GZH1	4.45 ± 6.79 × 10^−6^	−0.43 ± 0.076
GZH2	1.94 ± 3.29 × 10^−7^	−0.39 ± 0.107
GZH3	4.93 ± 4.24 × 10^−7^	−0.45± 0.115

**Table 5:** The corrosion current density and corrosion voltage of each group of samples after being immersed in SBF for 4 d.

Samples	Icorr (A/cm^2^)	Ecorr (V vs. Ag/AgCl)
Ti	1.77 ± 2.51 × 10^−6^	−0.42 ± 0.124
MAO	2.00 ± 3.01 × 10^−7^	−0.63 ± 0.155
HA	7.41 ± 9.51 × 10^−6^	−0.50 ± 0.187
GZH1	1.01 ± 1.66 × 10^−7^	−0.52 ± 0.103
GZH2	0.99 ± 1.39 × 10^−8^	−0.40 ± 0.076
GZH3	6.66 ± 4.70 × 10^−8^	−0.50 ± 0.046

**Table 6:** Correlation between cell proliferation rate and cytotoxic reaction rating.

Level	Relative Proliferation Rate
0	≥100
1	80–99
2	50–79
3	30–49
4	0–29

## Data Availability

The data are contained within the article.
